# Supplementary material for: Thermosensitive Drug Delivery System SBA-15-PEI for Controlled Release of Nonsteroidal Anti-Inflammatory Drug Diclofenac Sodium Salt: A Comparative Study
Source: Materials (Basel). 2021 Apr 9;14(8):1880. doi: 10.3390/ma14081880 (PMC8068836; doi:10.3390/ma14081880)
Supplement: Supplementary file 1 [file materials-14-01880-s001.zip › materials-1143976-supplementary.pdf]

materials

Supplementary Materials

Thermosensitive Drug Delivery System SBA-15-PEI for Controlled Release of Nonsteroidal Anti-inflammatory Drug Diclofenac Sodium Salt: A Comparative Study

Lubos Zauska [1], Stefan Bova [2], Eva Benova [1], Jozef Bednarcik [3], Matej Balaz [4], Vladimir Zelenak [1], Virginie Hornebecq [5] and Miroslav Almasi [1,*]

[1] Department of Inorganic Chemistry, Faculty of Science, P. J. Šafárik University, Moyzesova 11, SK-041 01 Košice, Slovakia; lubos.zauska@student.upjs.sk (L.Z.); evapopjakova@gmail.com (E.B.); vladimir.zelenak@upjs.sk (V.Z.)
[2] BovaChem s.r.o, Garbiarska 1919/14, SK-048 01, Rožňava, Slovakia; stefan.bova@gmail.com
[3] Institute of Experimental Physics, Slovak Academy of Sciences, Watsonova 47, SK-040 01 Košice, Slovakia; jozef.bednarcik@upjs.sk
[4] Institute of Geotechnics, Slovak Academy of Sciences, Watsonova 45, SK-040 01 Košice, Slovakia; balazm@saske.sk
[5] Aix-Marseille University, CNRS, MADIREL, F-133 97 Marseille, France; virginie.hornebecq@univ-amu.fr
* Correspondence: miroslav.almasi@upjs.sk; Tel.: +421 552-342-366

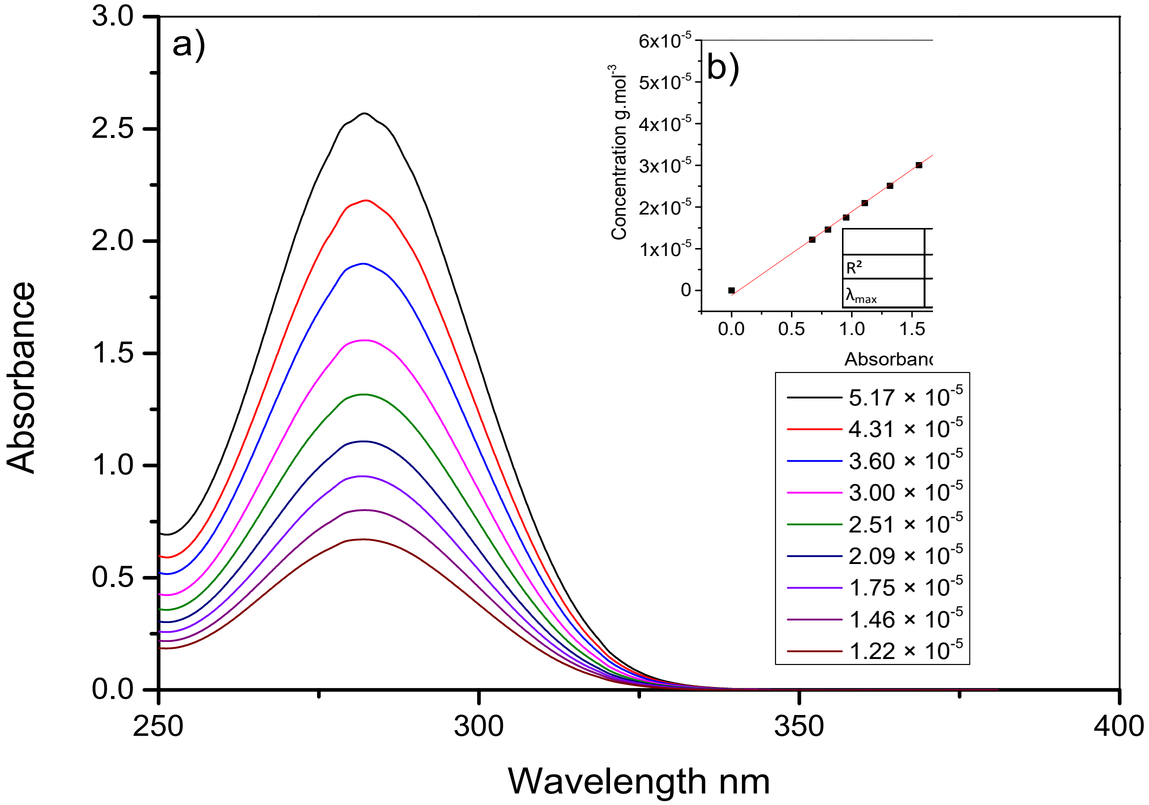

Figure S1. (**a**) UV spectra of diclofenac sodium methanolic solutions and (**b**) corresponding calibration curve.

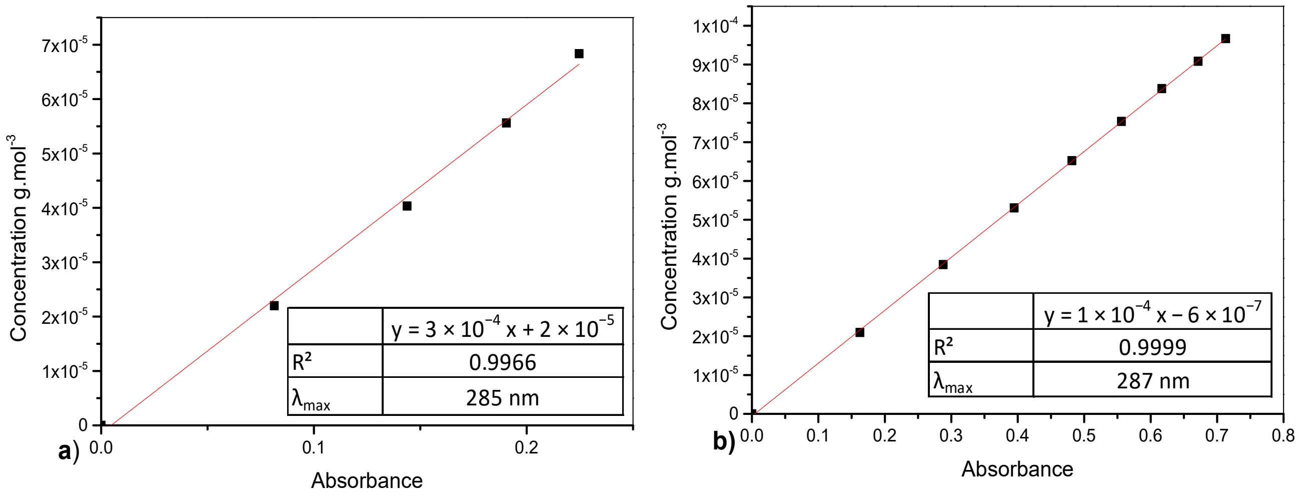

Figure S2. Calibration curve of diclofenac sodium in saline solutions at (**a**) pH = 2 and (**b**) pH = 7.4.

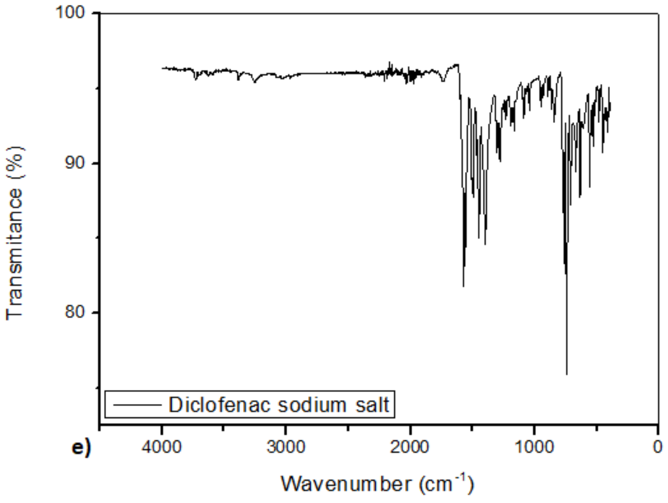

Figure S3. IR spectum of diclofenac sodium salt.

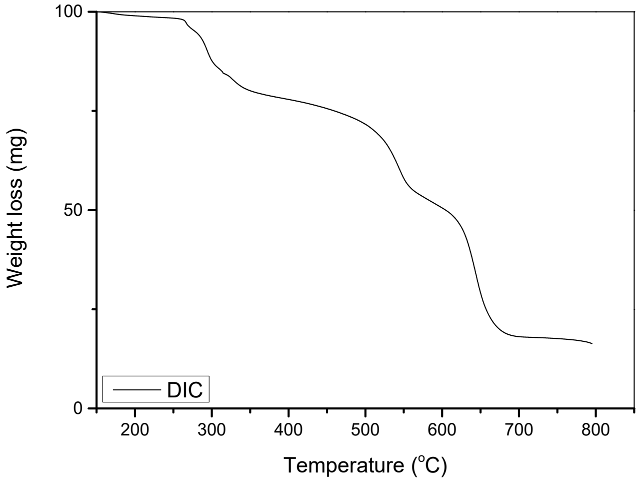

Figure S4. Thermogravimetric curve of diclofenac sodium salt.
